# In Vitro Effect of Copper (I) Complex [Cu(NN_1_)_2_](ClO_4_) on *Vibrio harveyi* BB170 Biofilm Formation

**DOI:** 10.3390/microorganisms9112273

**Published:** 2021-10-31

**Authors:** Sarita Soto-Aguilera, Brenda Modak, Maialen Aldabaldetrecu, Carla P. Lozano, Juan Guerrero, Claudia Lefimil, Mick Parra

**Affiliations:** 1 Laboratory of Biochemistry and Oral Biology, Faculty of Dentistry, Institute of Research in Dental Sciences, University of Chile, Olivos 943, Independencia, Santiago 8380492, Chile; sarita.soto@usach.cl (S.S.-A.); clozano@odontologia.uchile.cl (C.P.L.); 2 Laboratory of Natural Products Chemistry, Department of Environmental Sciences, Faculty of Chemistry and Biology, University of Santiago of Chile, Av. Bernardo O’Higgins 3363, Santiago 9170022, Chile; brenda.modak@usach.cl; 3 Aquaculture Biotechnology Center, Faculty of Chemistry and Biology, University of Santiago of Chile, Av. Bernardo O’Higgins 3363, Santiago 9170022, Chile; 4 Laboratory of Coordination compounds and Supramolecularity, Department of Materials Chemistry, Faculty of Chemistry and Biology, University of Santiago of Chile, Av. Bernardo O’Higgins 3363, Santiago 9170022, Chile; maialen.aldabaldetrecu@usach.cl (M.A.); juan.guerrero@usach.cl (J.G.)

**Keywords:** biofilm, antibacterial activity, antibiofilm activity, copper (I) complex, coumarin

## Abstract

Biofilm formation in pathogenic bacteria is an important factor of resistance to antimicrobial treatments, allowing them to survive for a long time in their hosts. In the search for new antibiofilm agents, in this work we report the activity of a copper (I) complex, [Cu(NN_1_)_2_]ClO_4_, synthesized with Cu (I) and NN1, an imine ligand 6-((quinolin-2-ylmethylene)amino)-2H-chromen-2-one, a derivate of natural compound coumarin. The antibacterial and antibiofilm capacity was evaluated in *Vibrio harveyi* BB170 used as model bacteria. Antibacterial activity was measured in vitro by minimal inhibitory concentration (MIC), minimal bactericidal concentration (MBC) and half-maximal inhibitory concentration (IC_50_) determination. Antibiofilm capacity of copper (I) complex was analyzed by different concentrations of IC_50_ values. The results showed that the sub-IC_50_ concentration, 12.6 µg/mL of the copper (I) complex, was able to reduce biofilm formation by more than 75%, and bacterial viability was reduced by 50%. Inverted and confocal laser scanning microscopy showed that the [Cu(NN_1_)_2_]ClO_4_ complex affected the biofilm structure. Therefore, the copper (I) complex is effective as an antibiofilm compound in *V. harveyi* BB170.

## 1. Introduction

Microorganisms can occur in two forms, planktonic or sessile [1]. Biofilm corresponds to the sessile estate and is developed from microbial adhesion to a surface followed by the formation of cell clusters or microcolonies that mature and stabilize in a matrix structure of extracellular polymeric substance (EPS), composed of polysaccharides, proteins and extracellular DNA (eDNA) [2]. The EPS structure provides protection against environmental stressors, decreasing microbial susceptibility to antimicrobial treatments compared to its planktonic form [1,3,4,5].

The formation of biofilms by bacterial pathogens is involved in over 80% of bacterial infections in many environments [5]. For example, *Pseudomonas aeruginosa* is able to colonize the human lungs and form biofilm, which does not respond to antibiotic treatment and causes persistent infections [6]. In the food industry, the presence of biofilm forming bacteria *Listeria monocytogenes* and *Salmonella* spp. causes contamination in food processing equipment, consequently bringing contamination and significant gastrointestinal infections in humans [7]. In marine environments, biofilm forming bacteria such as *Vibrio* spp. are responsible for the high mortality of aquaculture marine organisms in natural populations and farmed stocks around the world, affecting mainly crustaceans, mollusks and teleost fishes [8,9,10,11]. Within this genus, *Vibrio harveyi*, a Gram-negative luminescent bacteria, uses the formation of biofilm to infect and remain in their hosts [10], causing several diseases in shrimp, such as enteric vibriosis, appendix and cuticular vibriosis, localized wound vibriosis, shell disease, systemic vibriosis, septic hepatopancreatitis and tail rot disease [12]. In fishes, this disease includes vasculitis, gastroenteritis and eye lesions [11].

These examples show the effect of biofilm formation in different infectious diseases in both humans and animals, and it is essential to develop effective treatments that can reduce or inhibit the biofilm formation. In this sense, plants are an abundant source of bioactive molecules; among them are Coumarins, which have activity against a large number of bacterial strains, including multi-resistant bacteria [13]. Coumarins have also shown an effect on the biofilm formation inhibition of various bacteria, including *Escherichia* coli O157: H7, *Staphylococcus aureus*, *P. aeruginosa* and *Salmonella* Typhimurium [14,15,16,17].

In addition, some metals have been used as antimicrobials, such as copper, which inhibits the growth of various bacteria such as methicillin-resistant *S.* *aureus* (MRSA), *Clostridioides difficile*, *E.* *coli* and *Legionella pneumophila* [18]. The antibiofilm effect of copper has been described in several bacteria, for example *Salmonella* Enteritidis and *S. aureus* [17,19]. Similarly, metallic copper has shown effectiveness as an antimicrobial surface, and use of this metal in conjunction with disinfectant substances improves its activity, effectively eliminating biofilms on *P. aeruginosa* [13,20].

Considering the antibacterial properties of both natural compounds and copper, the effect of copper complexes with natural ligands has been studied, and a better antibacterial effect of the complex than its precursors has been observed [21,22,23]. On the other hand, the use of copper as a metallic center of the complex has shown to have a better antibacterial effect than other metals [24]. However, it has not been analyzed whether the antibiofilm capacity of these complexes is more effective.

**Figure 1:**
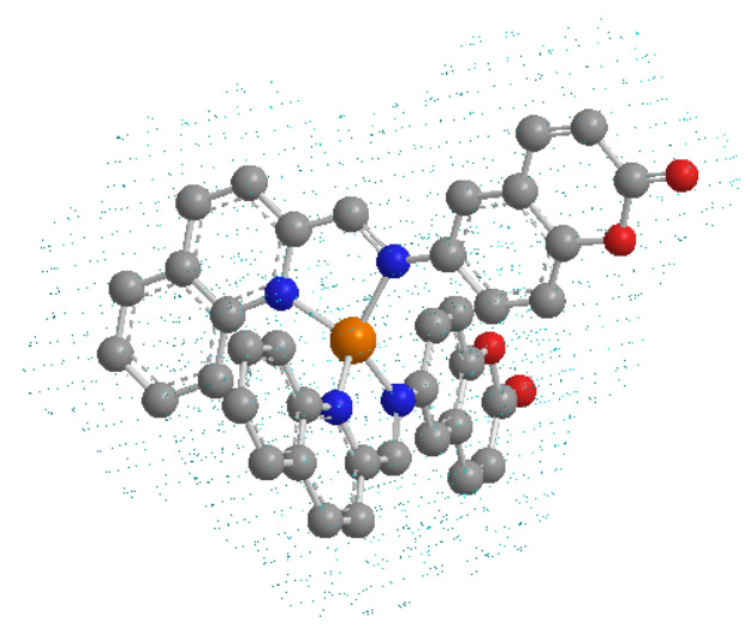
The structure of the copper (I) complex, [Cu(NN_1_)_2_]ClO_4_, used for the study. Orange, copper atom; blue, nitrogen atoms; red, oxygen atoms; gray, carbon atoms. Hydrogen atoms are omitted for a greater understanding of the molecule.

Recently, our group work showed the efficacy of a new copper (I) complex that combines coumarin derivate ligand and copper, forming a Cu (I) coordination complex [Cu(NN_1_)_2_]ClO_4_ (Figure 1), where NN_1_ is an imine ligand 6-((quinolin-2-methylene) amino)-2H-chromen-2-one obtained by a derivatization of natural compound coumarin. It inhibits the growth of the marine pathogen *Flavobacterium psychrophilum* [25]. Its antibacterial activity was greater than its components separately. However, the antibiofilm capacity of this compound has not been addressed.

Considering the abovementioned studies, the present work evaluated the effect of the coordination [Cu(NN1)_2_]ClO_4_ complex (copper (I) complex) on the ability to inhibit bacterial biofilm formation using the bacterium *V. harveyi* BB170 as a study model. The activity shown by its precursors coumarin and [Cu(CH3CN)_4_]ClO_4_ (copper (I) salt) was also investigated.

## 2. Materials and Methods

### 2.1. Chemical Compounds

Copper (I) complex, [Cu(NN_1_)_2_]ClO_4_, previously synthesized and characterized in solution by NMR techniques, UV–Vis and cyclic voltammetry [25], was used to test the effect on growth, biofilm formation and bacterial viability of *V. harveyi* BB170. Coumarin and copper (I) salt [Cu(CH_3_CN)_4_]ClO_4,_ precursors of the synthesis of the copper (I) complex, were used as compound effect controls. As an antibiotic effect control, oxytetracycline, was used in the bacterial growth assay, since it is one of the most commonly used antibiotics for the treatment of *Vibrio* spp. in aquaculture [26].

### 2.2. Bacterial Strain and Growth Conditions

*Vibrio harveyi* BB170 (obtained from the American Type Culture Collection, Manassas, VA, USA), was cultured aerobically in AB medium for 48 h at 30 °C [27].

### 2.3. Measurement of Growth of V. harveyi BB170

Effect of copper (I) complex, coumarin and copper (I) salt on growth of *V. harveyi* BB170 was evaluated by the minimal inhibitory concentration (MIC), minimal bactericidal concentration (MBC) and half-maximal inhibitory concentration (IC_50_). The MIC was determined using a microtiter plate assay as described [28], with some modifications. For this purpose, *V. harveyi* BB170 was grown in AB broth at 30 °C, 100 rpm and with aeration for 24 h with serial dilutions of compounds from 1024 to 0 µg/mL. *V. harveyi* BB170 without compounds was used as a positive growth control (untreated control). Oxytetracycline was used at the same concentrations of the compounds and AB broth without cells as a negative control. The OD_600_ nm was registered by spectrophotometry (Synergy HT Microplate Reader, BioTek^®^, Winooski, VT, USA). The lowest compound concentrations that inhibited bacterial growth by visual comparison with the negative control were considered as the MIC. The MBC determination was carried out following the protocol [29], with some modifications. An aliquot of 5 μL obtained from final cultures of MIC determination was grown on AB agar plates at 30 °C for 24 h. MBC corresponds to the lowest concentration of the compounds able to reduce the bacterial colony count to 99.9%. Bacterial cultures were performed from the MIC dilutions of each compound. For the determination of the half-maximum inhibitory concentration (IC_50_) of compounds in *V. harveyi* BB170, a correlation of OD_600_ nm measured of each compound concentration was made with AB medium without inoculating. It was subtracted from OD_600_ nm with grown *V. harveyi* BB170 with each compound concentration, obtaining the final OD. The concentrations were transformed into log (10) and OD_600_ nm and normalized in percentage. For the calculation of IC_50_, non-linear regression was performed with the GraphPad Prism version 5 program (GraphPad Software, San Diego, CA, USA).

### 2.4. Biofilm Quantification Assay

The effect of the IC_50_ values of the compounds on biofilm formation was analyzed. We analyzed the effect of the compounds on early (0–48 h) and mature (48–72 h) biofilm. For this, the IC_50_ values, two times the concentration of IC_50_ (2× IC50), half the concentration of IC_50_ (IC50/2) and a quarter of IC_50_ (IC50/4) of each compound were used in order to determine dose-dependent effect. *V. harveyi* BB170 was inoculated in 96-well plates at OD_600_ nm 0.05 and corresponding concentrations to IC_50_ evaluated. To determine the effect on early biofilm stage, the compounds were added at 0 h, and biofilm biomass was measured at 48 h. To determine the effect on mature biofilm, the compounds were added at 48 h, and biofilm biomass was measured at 72 h. The cultures were grown at 30 °C without shaking. Biofilm quantification was carried out by crystal violet (CV) assay as described [30], and the absorbance was measured by spectrophotometry, OD_585_ nm, using a microplate reader (Synergy HT Microplate Reader, BioTek^®^, Winooski, VT, USA).

Subsequently, to evaluate whether the concentration of copper (I) complex that showed the greatest inhibition of biofilm formation was a compound specific effect, this same concentration for coumarin and copper (I) salt was tested. Cultures of *V. harveyi* BB170 were carried out in 96-well plates at OD_600_ nm, 0.05 with each compound and incubated at 30 °C for 48 h without shaking. The biofilm formation was analyzed by CV staining as explained above.

### 2.5. Bacterial Biofilm Viability

Bacterial viability in the *V. harveyi* BB170 biofilm was evaluated with the copper (I) complex concentration with the highest inhibition of biofilm formation. For this purpose, *V. harveyi* BB170 was grown in 96-well plates at OD_600_ nm, 0.05, and copper (I) complex, coumarin and copper (I) salt were added in equal concentration and incubated at 30 °C for 48 h without shaking. Subsequently, the MTT assay reaction was carried out to determine viability of the bacteria forming the biofilm. Cell Proliferation Kit I (Roche Diagnostics, Meylan, France) was used to perform the test according to the supplier’s instructions. The absorbance resulting from the MTT assay at OD _550_ nm was recorded by the microplate reader (Synergy HT Microplate Reader, BioTek^®^, Winooski, VT, USA).

### 2.6. Biofilm Observation by Microscopy

To observe the effect of the copper (I) complex on the biofilm structure, microscopy analyses were performed. *V. harveyi* BB170 biofilm formation was carried out on glass slides (1 × 1 cm) placed in a 24-well plate with 500 µL of AB medium, bacterial inoculum OD_600_ nm_,_ 0.05, and compounds added in equal concentration and incubated for 48 h at 30 °C.

For inverted microscopy analysis, the glass slides were washed three times with 1× PBS, and the biofilm was fixed with methanol for 10 min and stained with 0.1% CV for 5 min. The CV excess was removed, and each slide was washed three times with distilled water, allowed to dry at room temperature and observed under an inverted microscope (Motic AE 2000, Motic, Hong Kong, China) at a magnification of 400×. This analysis makes it possible to differentiate between extracellular polymeric substance (EPS) formation and bacteria (bacterial coaggregation).

For the confocal laser scanning microscopy (CLSM) assay, the glass slides with the grown biofilm were washed three times with distilled water, and the biofilm (extracellular polymeric substance, EPS) was stained with FilmTracer™ SYPRO™ Ruby Biofilm Matrix Stain 1× (Invitrogen, Waltham, MA, USA) for 1 h at 4 °C. Previously the glass slides had been washed twice with distilled water to remove excess staining solution and placed on a microscope slide with one drop of 100 mg/mL DABCO (1,4-diazabicyclo [2.2.2.] octane) (Sigma, Darmstadt, Germany) and sealed with a few drops of acrylic enamel. The biofilm images were acquired in a confocal microscope equipped with a solid-state laser (CLSM) (LSM 800, Carl Zeiss, Jena, Germany). Optics used were Plan-Apochromat 40×/1.4 Oil DIC (UV) VIS-IR M27, objective laser excitation 561 nm at 0.50% and energy transmission 488 nm at 75%. The Z-stack analysis of surface topography and 3D architecture of the biofilm as well as the other CLSM images was carried out with Zen 2.6 software (Carl Zeiss, Jena, Germany).

## 3. Results

### 3.1. Antibacterial Effect of Compounds on V. harveyi BB170 Growth

**Figure 2:**
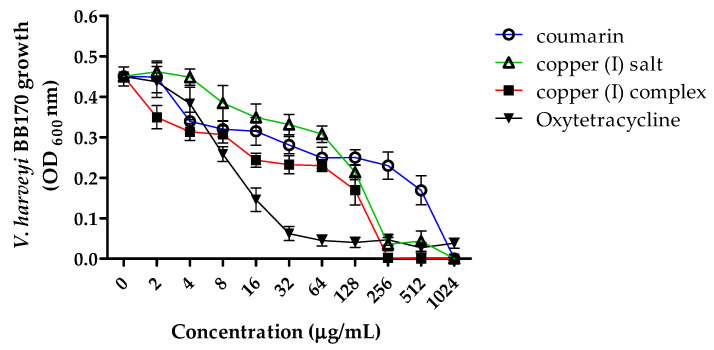
Effect of the different compounds on *V. harveyi* BB170 growth. Bacterial growth with 0–1024 µg/mL of coumarin, copper (I) complex, copper (I) salt and Oxytetracycline (antibiotic control). 0 µg/mL represents untreated control. The results show absorbance values from the bacterial culture measured to 24 h (OD _600_ nm). Three independent replicates.

Bacterial growth was performed for 24 h with serial dilutions from 1024 to 2 µg/mL of copper (I) complex and its precursors coumarin and copper (I) salt. The results revealed differences in the bacterial growth according to different compound treatments with respect to the untreated control (0 µg/mL) (Table S1 shows the statistical values from Figure 2). The copper (I) complex produced lower bacterial growth than coumarin and copper (I) salt at all the concentrations analyzed, being statistically significant with respect to the untreated control from the concentration of 32 to 1024 µg/mL. Although the highest concentrations (256–512–1024 µg/mL) of the three compounds showed a statistically significant decrease in bacterial growth (*p* < 0.05), the decrease was greater for copper (I) complex, where the lowest OD values were observed, reaching 0.0, being lower than those observed with oxytetracycline (antibiotic control) (Figure 2). *V. harveyi* BB170 growth curves with each compound from 0 to 24 h are shown in Figure S1.

Regarding the concentration of each compound necessary to inhibit and kill the bacteria (MIC and MBC), the results indicated a MIC value for copper (I) complex and copper (I) salt 4 times lower than coumarin. On the other hand, the MBC value for copper (I) complex was 2 times lower than for copper (I) salt and 4 times lower than for coumarin (Table 1). The analysis of IC_50_ showed the lowest value for copper (I) complex, being 0.28 times the IC_50_ value observed for coumarin (89.38 µg/mL) and 0.35 for copper (I) salt (71.9 µg/mL), indicating that approximately 4 times less copper (I) complex (25.19 µg/mL) is required to produce the same antibacterial effect on *V. harveyi* BB170 (Table 1).

### 3.2. Antibiofilm Effect of Compounds in V. harveyi BB170

Prior to this study, we determined the culture conditions and times in which the *V. harveyi* BB170 biofilm is formed. At 24 h no biofilm formation was observed, at 48 h a weak biofilm formation was observed while at 72 h a strong biofilm formation was observed (data not shown). For this reason, 0–48 h was considered as early biofilm and 48–72 h as mature biofilm.

**Figure 3:**
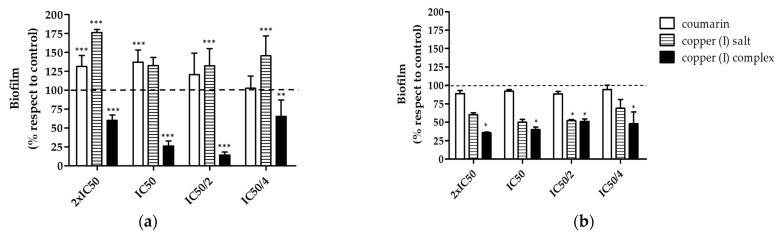
Effect of the compounds on *V. harveyi* BB170 biofilm formation. The effect of coumarin, copper (I) salt and copper (I) complex were analyzed in two stages of biofilm formation: early biofilm (0–48 h) and mature biofilm (48–72 h). The bacteria were grown in vitro with concentrations corresponding to IC_50_, 2X IC_50_, IC_50_/2, IC_50_/4 for each compound. (**a**) Effect on early biofilm. Compounds were added to *V. harveyi* BB170 culture at 0 h, and biofilm was measured 48 h after bacterial growth. (**b**) Effect on mature biofilm. Compounds were added to *V. harveyi* BB170 culture at 48 h, and biofilm was measured at 72 h. Crystal violet absorbance (OD_585_ nm) from each biofilm stage was normalized with respect to untreated control. Line represents untreated control value (100%). Statistical analysis, *t*-test, * *p* < 0.05, ** *p* < 001, *** *p* < 0.001. Three independent replicates.

IC_50_ values of each compound were used to analyze their effect on early and mature *V. harveyi* BB170 biofilm (Table 2). In early biofilm (0–48 h), treatment with copper (I) complex showed a statistically significant decrease (*p* < 0.05) in biofilm formation at all concentrations tested with respect to untreated control. The highest reduction in biofilm biomass occurred with 12.6 µg/mL (IC50/2), reducing the amount of biofilm biomass by 80% with respect to the untreated control. The effect of decreasing the formation of the biofilm begins to be lost with 6.3 µg/mL (IC50/4, Table 2), due to the low concentration of copper (I) complex. On the other hand, coumarin showed an increase of up to 53% of the biofilm biomass formed with 178.76 µg/mL (2× IC50) with respect to the untreated control. In addition, copper (I) salt showed an increase in the amount of biofilm formed, which reached 79% more than the untreated control with 143.8 µg/mL (2× IC50) (Figure 3a). When analyzing the effect on mature biofilm (48–72 h), coumarin did not show statistically significant variation in relation to the untreated control. Copper (I) complex induced a significant decrease, which reached 55% with 50.38 µg/mL (2× IC50), and 40% and 50% with 12.6 (IC50/2) and 6.3 µg/mL (IC50/4), with respect to the untreated control (Figure 3b). Copper (I) salt significantly decreased biofilm formation by 50% with 35.95 µg/mL (IC50/2). The results show that copper (I) complex notably affected the development of the biofilm in the early stage, specifically with 12.6 µg/mL, and in less quantity in the mature biofilm. However, in both states this decrease was greater than for coumarin and copper (I) salt. A dose-dependent response to the concentration of the evaluated compounds was not observed.

**Figure 4:**
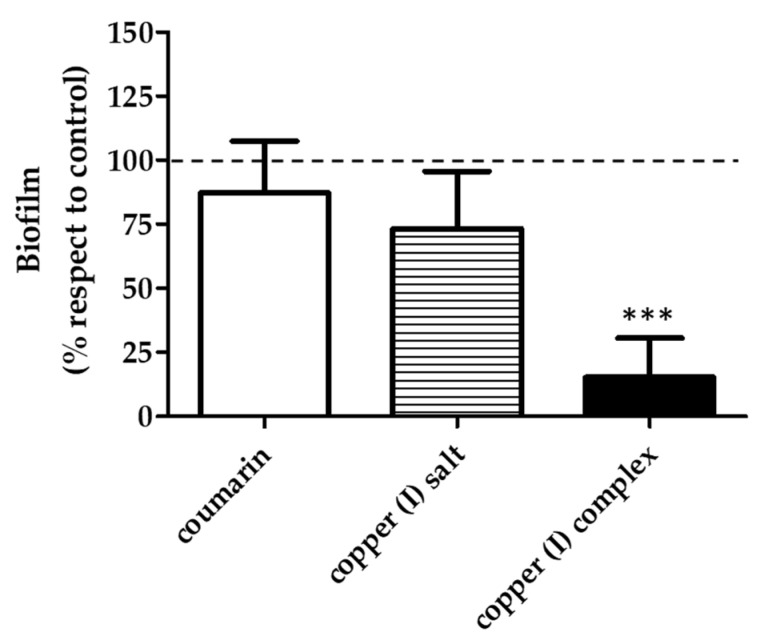
Effect of each compound at 12.6 µg/mL final concentration (IC_50_/2 value corresponds to copper (I) complex on *V. harveyi* BB170 biofilm formation). The biofilm formation was measured at 48 h. Crystal violet absorbance (OD_585_ nm) of each treatment was normalized with respect to the untreated control. Line represents untreated control value (100%). Statistical analysis, *t*-test, *** *p* < 0.001. Three independent replicates.

Since the highest effect on the decrease in the biofilm biomass was observed when evaluating the early biofilm with the concentration corresponding to copper (I) complex IC_50_ /2 (12.6 µg/mL), a test was conducted to assess whether this effect was caused by copper (I) complex specifically. For this, the effect on early biofilm formation of the three compounds at the same concentration (12.6 µg/mL) was tested. The results show that copper (I) complex decreased the biofilm biomass by 75% with respect to the untreated control, while the coumarin and copper (I) salt did not show differences in biofilm development with respect to the untreated control (Figure 4). Therefore, the greatest inhibitory effect on the development of the biofilm of *V. harveyi* BB170 observed (Figure 3a) was a result of the specific concentration of the copper (I) complex.

### 3.3. Effect of the Compounds on Bacterial Viability

**Figure 5:**
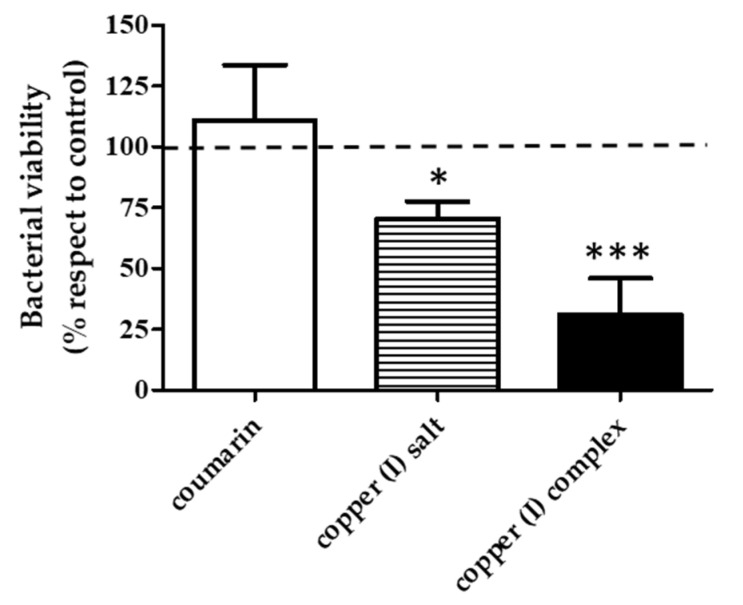
Effect of the compounds on bacterial biofilm viability in *V. harveyi* BB170. Bacteria was grown for 48 h with compounds (12.6 µg/mL each one), and the MTT assay was performed on the biofilm formed. MTT absorbance (OD_550_ nm) was measured in each treatment and normalized with respect to the untreated control. Line represents untreated control value = 100%. Statistical analysis, *t*-test, * *p* < 0.05, *** *p* < 0.001.Three independent replicates.

The next step was to evaluate the effect of each compound at 12.6 µg/mL on *V. harveyi* BB170 biofilm bacterial viability. For this, an MTT test was performed on the biofilm treated with the compounds at the mentioned concentration. The copper (I) complex produced a 50% reduction in bacterial viability, and the copper (I) salt reduced bacterial viability by 25% compared to the untreated control, while coumarin did not show an effect on the viability of the bacterial biofilm with respect to the untreated control (Figure 5).

### 3.4. Effect of the Compounds on V. harveyi BB170 Biofilm Structure

**Figure 6:**
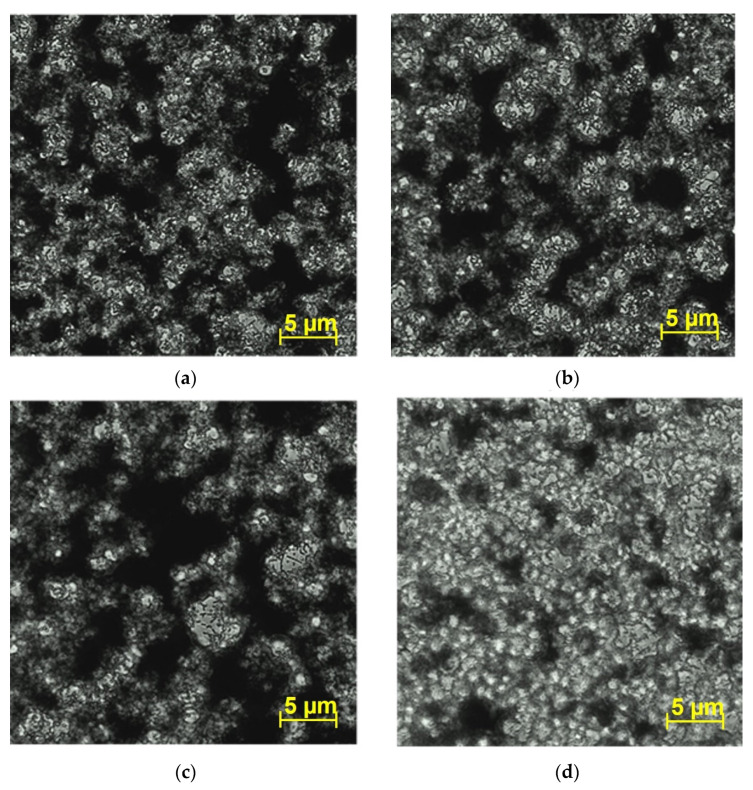
Effect of compounds on biofilm formation observed by the inverted microscope. In vitro *V. harveyi* BB170 biofilm formation treated with 12.6 µg/mL of each compound for 48 h. (**a**) Untreated control, (**b**) coumarin, (**c**) copper (I) salt, (**d**) copper (I) complex. 400×. Dark zones: extracellular polymeric substance (EPS). Clear zones: bacterial coaggregation.

In order to analyze the effect of copper (I) complex on *V. harveyi* BB170 biofilm architecture, the biofilm formed on 48 h cultures treated with 12.6 µg/mL of each compound was observed by inverted microscopy (Figure 6). Microscopic analysis of the untreated control showed a biofilm with abundant extracellular polymeric substance (EPS), seen as the dark areas (Figure 6a). However, the bacteria treated with the copper (I) complex showed a poor development of EPS, with fewer dark areas and greater quantity of bacterial coaggregates observed, seen as the clear areas in the microscope image (Figure 6d). With coumarin (Figure 6b) and copper (I) salt (Figure 6c) treatments, no major differences were observed in the EPS structure with respect to untreated control (Figure 6a).

**Figure 7:**
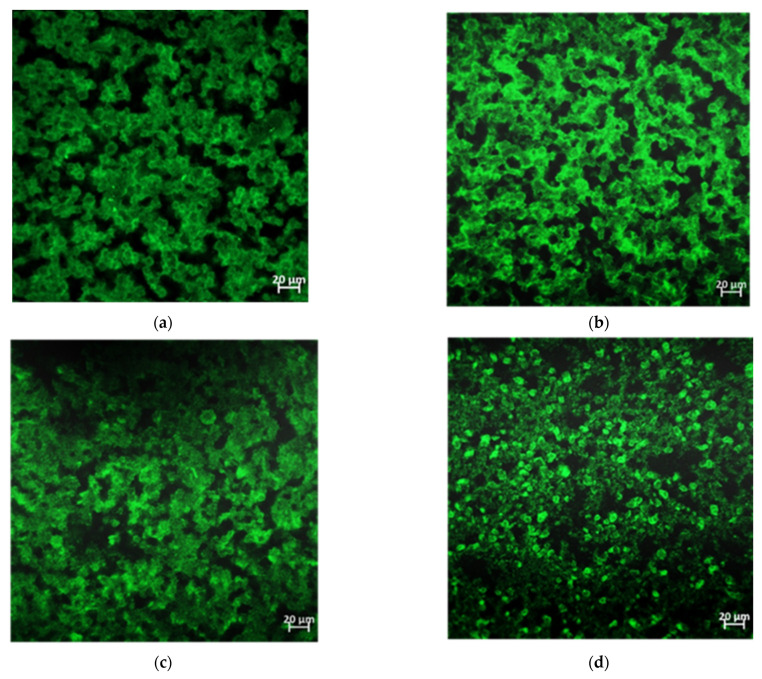

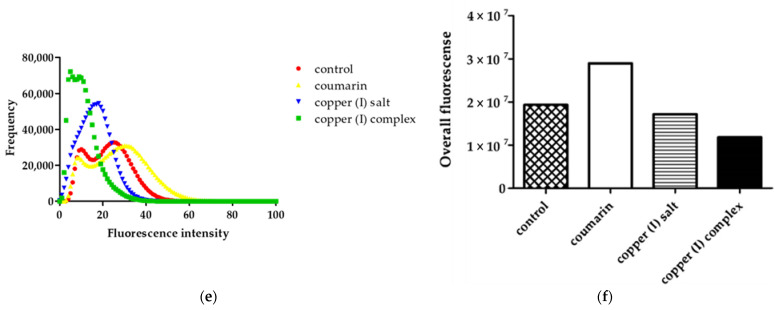
Effect of compounds on biofilm formation observed by CLSM. In vitro *V. harveyi* BB170 biofilm formation treated with 12.6 µg/mL of each compound for 48 h.(**a**) Untreated control, (**b**) coumarin, (**c**) copper (I) salt, **(d)** copper (I) complex. Extracellular polymeric substance (EPS) was marked using FilmTracer ™ SYPRO ™ Ruby Biofilm Matrix Stain. (**e**) *V. harveyi* BB170 biofilm fluorescence intensity from CLSM images (**a**–**d**), (**f**) overall fluorescence from CLSM images (**a**–**d**).

To confirm the modifications in the biofilm architecture observed by inverted microscopy, an analysis of the biofilm of *V. harveyi* BB170 was also carried out by means of confocal laser scanning microscopy (CLSM). It showed similar results because the biofilms formed under coumarin and copper (I) salt treatment have large and compact green areas of EPS structures (Figure 7a–c). In contrast, the structure of *V. harveyi* BB170 biofilm formed in the presence of copper (I) complex has smaller green areas and heterogeneous EPS structure compared to the untreated control (Figure 7d), indicating a variation in the biofilm structure caused by the copper (I) complex. To further analyze whether these changes affected the amount of EPS, a fluorescence intensity curve of the formed *V. harveyi* BB170 biofilm was performed. The results showed that the biofilm formed under coumarin treatment had a mean fluorescence intensity (MFI) slightly higher than that observed in the case of the untreated control (28 and 23 MFI, respectively). On the other hand, the biofilm formed with copper (I) salt has a mean fluorescence intensity lower than the untreated control (16 and 23 MFI, respectively). Furthermore, the biofilm formed with copper (I) complex has a mean fluorescence intensity even lower than the untreated control (10 and 23 MFI, respectively) (Figure 7e).

In addition, the total fluorescence of the area of the biofilm grown under each compound was measured, and it was observed that the biofilm treated with the copper (I) complex showed a lower fluorescence than the biofilm treated with coumarin and copper salt and a lower fluorescence than the untreated control (Figure 7f). These results show that effectively the amount of EPS in the early *V. harveyi* BB170 biofilm with copper (I) complex decreased, which is consistent with the changes visually observed in its structure (Figure 6d and Figure 7d).

**Figure 8:**
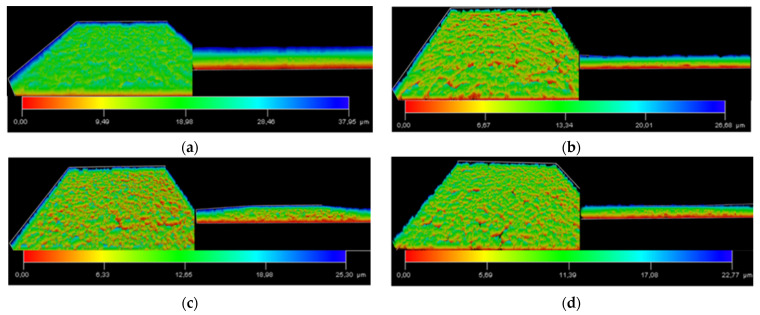
Effect of each compound on *V. harveyi* BB170 biofilm formation observed by CLSM. In vitro biofilm formation treated with 12.6 µg/mL of each compound for 48 h. (**a**) Untreated control, (**b**) coumarin, (**c**) copper (I) salt, (**d**) copper (I) complex.

Additionally, an analysis of the 3D architecture of the biofilm of *V. harveyi* BB170 formed with the different treatments was carried out (Figure 8). In the case of the untreated control, the biofilm has a thickness of 37.95 µm (Figure 8a). On the other hand, with the coumarin, copper salt and copper complex treatments, it decreased to 26.68 (Figure 8b), 25.30 (Figure 8c) and 22.77 µm (Figure 8d), respectively. These results show that the copper (I) complex is not only capable of reducing the biofilm biomass that *V. harveyi* BB170 produces, as previously observed (Figure 3a, Figure 6d and Figure 7d), but it also reduces the thickness of the biofilm with respect to the untreated control. On the other hand, coumarin and copper (I) salt also showed a decrease in the thickness of the biofilm; however, it was less than that observed for copper (I) complex.

## 4. Discussion

Biofilms formed by pathogenic bacteria constitute a challenge for conventional antimicrobial treatments, since in general, it is not possible to completely eliminate the infection, causing recurrent episodes of infection after antibiotic treatments [31,32]. In search of new and better alternatives to bacterial biofilms, in this work we analyzed the effect of the copper (I) complex [Cu(NN_1_)_2_]ClO_4_ (Figure 1) formed with Cu (I) and coumarin as ligands [25] on the *V. harveyi* BB170 biofilm. The effect on this bacterium was elucidated by measuring total planktonic growth, determining MIC, MBC, IC_50_ and evaluating the formation of biofilm through in vitro cultures with the presence of copper (I) complex and its precursors coumarin and copper (I) salt.

Our study revealed that copper (I) complex was more effective than coumarin and copper (I) salt in reducing the planktonic growth of *V. harveyi* BB170 in a 24-h period. For the three compounds analyzed, it was observed that the growth of *V. harveyi* BB170 decreased with respect to the untreated control. However, this decrease was greater in all concentrations evaluated by copper (I) complex_._ Furthermore, the IC_50_ copper (I) complex value was almost three times lower than that observed for coumarin and copper (I) salt, which shows that the copper (I) complex can more efficiently inhibit the growth of *V. harveyi* BB170. In line with these results, in a previous analysis carried out by our group, copper (I) complex reduced bacterial growth in *F. psychrophilum***,** as observed here, more effectively than its precursors, coumarin and copper (I) salt [25]. It has been proposed that this improved antibacterial effect shown by the copper (I) complex is a result of the chelation of Cu (I) by the coumarin derivate ligand, causing a partial shift of the positive charge through the metallic bond of the ligand [22,33,34]. This would favor the permeation of a greater amount of Cu (I) towards bacterial cells through the lipid membrane, affecting normal processes that take place in the microbial cell, generating the death of the bacteria [24,34]. In this sense, Cu (I) forming complexes with other molecules have shown to be capable of improving antibacterial capacity when compared to separate components [35].

On the other hand, when we analyzed the compound effects on the ability of *V. harveyi* BB170 to form biofilm, copper (I) complex showed its most relevant effect. Although the decrease in biofilm biomass by the copper (I) complex was observed in both states of the biofilm, the highest level of decrease was observed in the early stage of biofilm development with respect to the untreated control. Similar results were reported for a copper (II) complex using 2-thiouracil as a ligand, which decreases biofilm formation and destroys the preformed biofilm of *Candida albicans* [36]. This indicates that copper (I) complex would be more efficient in inhibiting the biofilm development than in eliminating the biofilm already formed in the mature state. While for copper (I) salt a reduction in the biomass of the mature biofilm was observed, this was less than that observed for copper (I) complex. In this sense, other studies with copper (II) complexes using ciprofloxacin as a ligand showed that the copper complexes decreased the biofilm formation of *P. aeruginosa* PAO1 more than free ciprofloxacin [37].

Furthermore, when testing sub-IC_50_ concentrations of the three compounds, copper (I) complex 12.6 µg/mL (IC_50_/2) reduced the production of early stage *V. harveyi* BB170 bacterial biofilm by 80%, with 50% viable bacteria decrease, while its precursors did not show this decrease at the same concentration analyzed. According to these findings, copper (I) complex was more efficient in biofilm reduction in the lowest concentrations, though this effect was diminished with IC50/4, presumably due to the higher dilution of copper (I) complex. However, it also maintained a greater effect than its precursors. This has also been observed in other studies with natural compounds where the greatest antibacterial effects are observed at lower concentrations of the compound [38,39].

Moreover, the results obtained from the effect of the copper (I) complex 12.6 µg/mL on the early biofilm showed that this complex was able to decrease the amount of biomass and affected its viability. These findings could indicate that the copper (I) complex disturbs the basis of the establishment of the bacterial biofilm of *V. harveyi* BB170 in its early stages of development, affecting the efficient adherence to the surface of the bacteria, a key step in the establishment of the biofilm and subsequent colonization of *V. harveyi* BB170 [40,41]. One possible way in which the copper (I) complex could affect the biofilm formation is by blocking the expression of specific proteins for bacterial attachment to the surface, thus preventing a sufficient quantity of bacteria from adhering to the surface to form a strong biofilm. Studies carried out with 4-phenyl coumarin demonstrated that this molecule was able to interfere with the adherence and formation of the biofilm of *S. aureus* [42]. Although in our study, coumarin did not decrease the formation of biofilms in *V. harveyi* BB170, the copper (I) complex did. Therefore, the formation of the copper (I) salt and coumarin in a complex coordination could favor the effect of both.

Furthermore, the copper (I) complex also affected the architecture and morphology of the early biofilm. Microscopy images showed that the copper (I) complex was able to decrease the amount of EPS matrix and the thickness of the biofilm formed more efficiently than coumarin and copper (I) salt. This fact is significant because in the biofilm structure, the EPS matrix provides stability to bacterial coaggregates and then gives way to a well-formed mature biofilm [43]. In this same sense, the copper (I) complex could affect eDNA, which is an important part of EPS and plays a key role in the initial adhesion and structural stability of the biofilm in Gram-negative and -positive bacteria [44]. Studies carried out in *Vibrio cholerae* have shown that eDNA is able to interact with the exopolysaccharides in the EPS, improving strength and stability of the biofilm and contributing to antimicrobial resistance [45]. In this respect, in analyses carried out in *P. aeruginosa* with copper (II) complexes, it has been shown that they are capable of increasing the antibiofilm effect by inhibiting the amount of biofilm eDNA [46]. However, studies in this regard are required in *V. harveyi* BB170 and copper (I) complex to evaluate these postulates.

On the other hand, it is worth mentioning that we observed an increase in the amount of biofilm formed with coumarin and copper (I) salt with respect to untreated control. Although we cannot fully explain these results, the effect observed with coumarin could be associated with its interaction with some molecules related to the increase in the EPS matrix biofilm production in *V. harveyi* BB170 [47,48,49]. The mechanisms underlying the interactions that coumarin could have with other molecules associated with the biofilm increase remain to be elucidated. Instead, the increase in biofilm formed after copper (I) salt treatment could be linked to some system of tolerance to copper that *V. harveyi* BB170 has overexpressed in the presence of ion Cu^+^, giving rise to the development of a greater amount of biofilm, but more studies are necessary on that subject.

Another possible way by which copper (I) complex could affect the biofilm formation of *V. harveyi* BB170 is through inhibition of the communication by quorum sensing (QS), where it is capable of establishing cell-to-cell communication in response to small molecules called autoinducers (AIs). They regulate the expression of different enzymes that give rise to several bacterial virulence factors [50,51,52,53,54]. Thus, the QS system has been described as an important cellular mechanism for biofilm establishment in many bacterial species [55]. In studies carried out in *P*. *aeruginosa*, copper (II) complexes were able to interfere with the QS signaling, also affecting the development of the bacterial biofilm [56]. Likewise, it has been shown that coumarin possesses antibiofilm capacity by inhibition of a wide range of molecules and autoinducers involved in QS, in bacteria such as *S. aureus, P. aeruginosa* and *E. coli* [15,16,57,58]. In our study it was not possible to observe an effect in the reduction of biofilm by coumarin. However, its effects could improve when forming the copper (I) complex and could affect QS. Accordingly, these proposed mechanisms could be part of the response observed with copper (I) complex and *V. harveyi* BB170 in this study, which will be analyzed in future work.

The results reported in this work on the copper (I) complex could demonstrate that the synthesis of copper (I) complexes using natural compounds that have previously shown antibiofilm activity as ligands could enhance the antibiofilm effect. On the other hand, in future work it is necessary to address whether the effect of this copper (I) complex is better than other reported copper complexes [36,37,59].

## 5. Conclusions

This study is the first report of antibiofilm activity of copper (I) complex [Cu(NN_1_)_2_]ClO_4_, with coumarin as a ligand, in the bacterial model *V. harveyi* BB170. It was shown to be effective in reducing the planktonic bacterial growth and reducing the biofilm formation compared with coumarin and copper (I) salt, where the presence of the Cu (I) ion forming the complex with coumarin would be fundamental for the observed antibiofilm effect. Accordingly, copper (I) complex would constitute a good candidate as an antibiofilm compound. However, studies elucidating the bacterial mechanisms affected by copper (I) complex to achieve this antibiofilm effect are yet to be conducted. In future works, it is necessary to analyze if the copper (I) complex can interfere with cell adherence, EPS production and QS in *V. harveyi* BB170 biofilm formation.

## Figures and Tables

**Table 1:** MIC, MBC, IC_50_ values of the coumarin, copper (I) salt, copper (I) complex in *V. harveyi* BB170. Different letters show statistically significant differences between treatments. Three independent replicates. Statistical analysis, *t*-test, *p* < 0.05.

Vibrio harveyi BB170			
	MIC µg/mL	MBC µg/mL	IC_50_ µg/mL
**Coumarin**	1024	1024	89.38 ± 0.05 ^a^
**Copper (I) salt** **[Cu(CH_3_CN)_4_]ClO_4_**	256	512	71.90 ± 0.02 ^a^
**Copper (I) complex** **[Cu(NN_1_)_2_]ClO_4_**	256	256	25.19 ± 0.04 ^b^

**Table 2:** IC_50_ values of copper (I) complex, coumarin and copper (I) salt.

IC_50_ (µg/mL)				
	2× IC_50_	IC_50_	IC_50_/2	IC_50_/4
**Coumarin**	178.76	89.38	44.69	22.35
**Copper (I) salt** **[Cu(CH_3_CN)_4_]ClO_4_**	143.8	71.9	35.95	17.97
**Copper (I) complex** **[Cu(NN_12_]ClO_4_**	50.38	25.19	12.6	6.3

## Data Availability

Not applicable.

## Supplementary Materials

The following are available online at www.mdpi.com/article/10.3390/microorganisms9112273/s1. Figure S1. Effect of the compounds in V. harveyi BB170 growth curve. Bacterial growth kinetics with serial dilutions from 1024 to 2 µg/mL of (a) coumarin, (b) copper (I) salt, (c) copper (I) complex, (d) oxytetracycline. The results show absorbance values from the bacterial culture measured every 2 h from 0 to 24 h (OD600 nm). Three independent replicates. Table S1. V. harveyi BB170 growth. Absorbance values (OD600 nm) of V. harveyi BB170 growth at 24 h with coumarin, copper (I) salt, copper (I) complex and oxytetracycline. Statistical analysis was conducted by comparing the bacterial growth of the treatments with the control at 24 h. t-test, * *p* < 0.05, ** *p* < 001, *** *p* < 0.001. Three independent replicates.
