# Supplementary material for: In Vitro Effect of Copper (I) Complex [Cu(NN1)2](ClO4) on Vibrio harveyi BB170 Biofilm Formation
Source: Microorganisms. 2021 Oct 31;9(11):2273. doi: 10.3390/microorganisms9112273 (PMC8618041; doi:10.3390/microorganisms9112273)
Supplement: Supplementary file 1 [file microorganisms-09-02273-s001.zip › microorganisms-1422427-supplementary.pdf]

Figure S1.

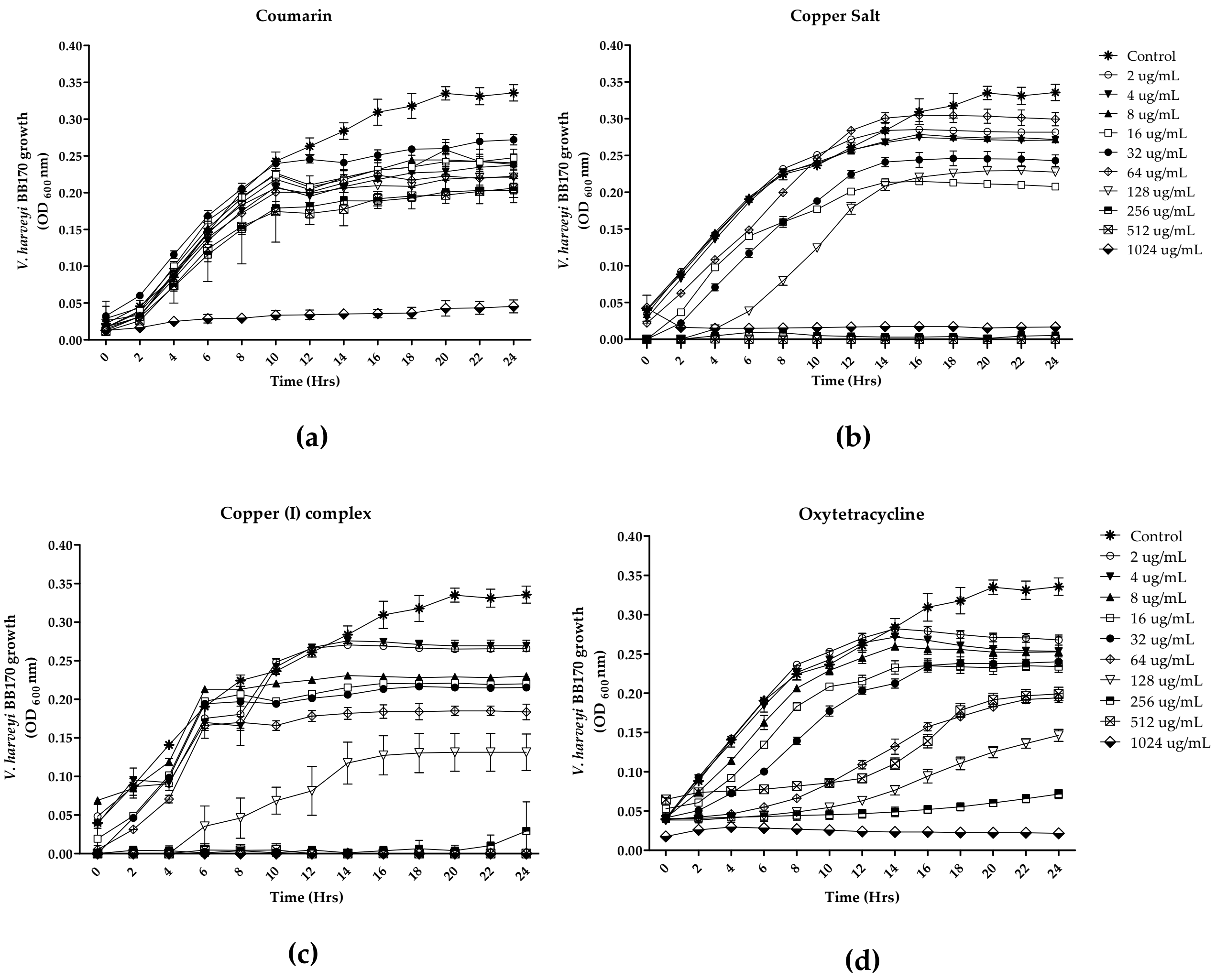

Figure S1. Effect of the compounds in *V. harveyi* BB170 growth curve. Bacterial growth kinetics with serial dilutions from 1024 to 2 μg/mL of **(a)** coumarin **(b)** copper (I) salt **(c)** copper (I) complex **(d)** Oxytetracycline. The results show absorbance values from the bacterial culture measured every 2 h from 0h until 24 h (OD_{600}nm). Three independent replicates.

Table S1. *V. harveyi* BB170 growth. Absorbance values (OD $_{600}$ nm) of *V. harveyi* BB170 growth at 24 h with coumarin, copper (I) salt, copper (I) complex and Oxytetracycline. Statistical analysis was made by comparing the bacterial growth of the treatments with the control at 24 h. T-test, **p* < 0.05, ***p* < 001, ****p* < 0.001. Three independent replicates.

**Concentration μg/mL**	**Absorbance values (OD 600 nm) of *V. harveyi* BB170**			
	**Coumarin**	**Copper salt $[Cu(CH_3CN)_4]ClO_4$**	**Copper (I) complex $[Cu(NN_1)_2]ClO_4$**	**Oxytetracycline**
**1024**	0.000 ± 0.000***	0.001 ± 0.003***	0.000 ± 0.000***	0.038 ± 0.013***
**512**	0.169 ± 0.036***	0.043 ± 0.025***	0.000 ± 0.000***	0.027 ± 0.013***
**256**	0.230 ± 0.033***	0.036 ± 0.016***	0.002 ± 0.005***	0.047 ± 0.013**
**128**	0.250 ± 0.019**	0.213 ± 0.018*	0.169 ± 0.036**	0.040 ± 0.012**
**64**	0.249 ± 0.026*	0.308 ± 0.020	0.230 ± 0.014*	0.045 ± 0.014**
**32**	0.281 ± 0.021	0.331 ± 0.025	0.232 ± 0.022*	0.062 ± 0.017*
**16**	0.315 ± 0.034	0.349 ± 0.033	0.243 ± 0.017	0.146 ± 0.029
**8**	0.320 ± 0.019	0.384 ± 0.043	0.307 ± 0.021	0.258 ± 0.018
**4**	0.339 ± 0.023	0.448 ± 0.020	0.313 ± 0.021	0.383 ± 0.040
**2**	0.449 ± 0.039	0.462 ± 0.022	0.349 ± 0.029	0.437 ± 0.038
**0**	0.450 ± 0.023	0.450 ± 0.023	0.450 ± 0.023	0.450 ± 0.023
